# Computational analysis of learning in young and ageing brains

**DOI:** 10.3389/fncom.2025.1565660

**Published:** 2025-05-06

**Authors:** Jayani Hewavitharana, Kathleen Steinhofel, Karl Peter Giese, Carolina Moretti Ierardi, Amida Anand

**Affiliations:** ^1^Department of Informatics, King's College London, London, United Kingdom; ^2^Department of Basic and Clinical Neuroscience, King's College London, London, United Kingdom; ^3^Department of Neuroimaging, King's College London, London, United Kingdom; ^4^Department of Physics, King's College London, London, United Kingdom

**Keywords:** ageing-brains, learning, memory, computational-neuroscience, neural networks

## Abstract

Learning and memory are fundamental processes of the brain which are essential for acquiring and storing information. However, with ageing the brain undergoes significant changes leading to age-related cognitive decline. Although there are numerous studies on computational models and approaches which aim to mimic the learning process of the brain, they often concentrate on generic neural function exhibiting the potential need for models that address age-related changes in learning. In this paper, we present a computational analysis focusing on the differences in learning between young and old brains. Using a bipartite graph as an artificial neural network to model the synaptic connections, we simulate the learning processes of young and older brains by applying distinct biologically inspired synaptic weight update rules. Our results demonstrate the quicker learning ability of young brains compared to older ones, consistent with biological observations. Our model effectively mimics the fundamental mechanisms of the brain related to the speed of learning and reveals key insights on memory consolidation.

## 1 Introduction

Learning is a fundamental process of the human brain, which is essential for acquiring, storing, and retrieving information as memories. Memory enables navigating the world and responding to various circumstances by making informed decisions. Moreover, it provides context for adapting to new situations based on past experiences and developing new skills over time. Learning enhances cognitive function by building neural connections between different pieces of information, which enhances critical thinking and problem-solving abilities. From a psychological point of view, memory has been described as the faculty for encoding, storing and retrieving information (Squire, 2009). It is how we navigate the world, build on past experiences, acquire new information and learn from it.

In an effort to understand the biological process of learning, Donald Hebb proposed a theory for the behavior of neural networks in the brain which is now commonly known as “Hebb's rule” (Hebb, 1949). Hebb's rule postulates that when two neurons are repeatedly activated simultaneously the synapse, or the connection between them, strengthens. This synpatic strengthening is thought to be the biological basis for learning and memory formation, and Hebb's theory laid the foundation for understanding the workings of synaptic plasticity, the fundamental mechanism by which the brain learns through strengthening and weakening neural connections.

Bliss and Lømo (1973) discovered long-term potentiation (LTP), which follows Hebb's postulate, and because of its long duration it is widely thought to underlie long-term memory. Moreover, various experimental evidence has also supported the idea that LTP underlies memory. This includes observations that indicate blocking LTP impairs memory, and learning induces LTP (Giese, 2012). It is also suggested by research which activate synapses with optogenetics that LTP induction is sufficient for memory formation (Nabavi et al., 2014). Researchers have observed that learning is induced in mice when activating synapses, and a different frequency of light stimulation that weakens the synapses blocks learning using a process called long-term depression (LTD). Moreover, there are morphological changes in dendritic spines that are associated with synaptic transmission which includes the formation and shrinkage of new dendritic spines (Ma and Zuo, 2022). Research by Bliss and Collingridge demonstrates the crucial role of N-methyl-D-aspartate (NMDA) receptors in initiating LTP, highlighting the molecular processes that support synaptic plasticity (Bliss and Collingridge, 1993). Further studies have shown that blocking NMDA receptors impairs LTP leading to deficits in spatial learning tasks (Nakazawa et al., 2004). Research also suggest that LTP and LTD are not unitary phenomena, but vary depending on the synapses and circuits involved allowing the brain to adapt to new experiences (Malenka and Bear, 2004). This indicates the importance of LTP and LTD in tasks that require the brain to remember spatial environments.

With the ageing population and the increase in rates of age-related neurological disorders, it is important to understand how learning differs between young and older individuals. Burke and Barnes (2006) discusses the age-related changes in synaptic plasticity and memory, emphasizing the decline in LTP and slower formation of new synaptic connections in older adults due to various changes in dendritic morphology, cellular connectivity, Calcium ion dysregulation, and gene expression. A study by Ménard et al. (2015) identified a correlation between the presynaptic and postsynaptic glutamatergic component expression in the hippocampus and spatial memory capacity, indicating that changes in receptor density in older brains may limit synaptic connectivity. A study by Lu et al. (2024) which aims to identify the differences in LTP-like plasticity between younger and older individuals discovered that LTP is reduced in older adults aged 60–80 resulting in lower performance in episodic memory, language function and global cognitive function. Additionally, theoretical models suggest that the number of memories that can be stored depends on the complexity of synaptic connections and the structure of representations stored in a neural network (Fusi, 2021). Findings from such models can be translated into biological systems, offering insights into the decline of memory in ageing brains.

Aziz et al. (2019) have looked at learning from a biological perspective in young and old mice and have found that the mechanism that seemed to lead to learning in old mice was not synaptic strength (LTP) but rather the increase in multi-innervated dendritic spines (MIS). This means that a dendritic spine with typically two excitatory inputs from different neurons is generated leading to the connection of three neurons (two presynaptic neurons and one postynaptic neuron). Studies with LTP-deficient mutant mice suggest that MIS generation requires more training trials than LTP, thus slowing down the speed of learning (Radwanska et al., 2011). Therefore, an age-related switch in learning mechanisms from LTP to MIS generation may explain in part the differences between learning in younger and older individuals.

To better understand biological systems, it is useful to view them through mathematical models. There are numerous different parameters to consider in biological brains, many that have not even been discovered yet; but in these models one usually tries to simplify them and select only a few that are relevant to the investigation. Therefore, they enable exploration of the complex biological mechanisms in a more controlled and isolated environment due to their simplified nature, offering insights that can be challenging to obtain by directly experimenting with living beings. For neurological experiments, a commonly used model of the brain is the artificial neural network (ANN), which is designed to simulate the structure and function of the brain's neural network. ANNs attempt to mimic the brain's ability to learn through mechanisms similar to synaptic plasticity to strengthen and weaken neural connections based on activity. Artificial neurons adjust the weights of their connections during learning which enables the network to recognize patterns and make predictions (Basheer and Hajmeer, 2000).

Structurally, ANNs consist of interconnected nodes arranged in layers. While there are ANNs that do not include hidden layers, many, such as multilayer perceptron (MLPs) and convolutional neural networks (CNNs) are feed-forward with hidden layers connoting that information only passes through the network in a single direction (Goodfellow et al., 2016). In such networks, connections between nodes are represented as weighted edges, and they determine how signals propagate through the network. Examples of ANNs used biologically can be seen in various studies, including (Shine et al., 2021), where models were used to link cellular mechanisms of neuromodulation to large-scale neural dynamics. They have created a model of the brain that represents cortical regions (boxes) that are thought to be organized into a functional hierarchy. A similar study has produced a model of the neurodegeneration from Alzheimer's disease (Jones et al., 2022).

Various other biologically inspired models have also been developed that specialize in specific computational learning tasks. Contrastive Hebbian learning (Xie and Seung, 2003) provides a biologically plausible mechanism for training energy-based neural networks by adjusting synaptic weights based on differences in network states. Hopfield networks (Hopfield, 1982) serve as classic models of associative memory, using recurrent connections to store and retrieve patterns. Restricted Boltzmann machines (RBMs) have also been widely used to model probabilistic representations of neural activity and learning dynamics (Hinton and Salakhutdinov, 2006). Although originally simple and abstract representations of basic neural function, brain-inspired models have evolved into highly sophisticated systems capable of deep learning and have shown success in tasks such as image-recognition, speech processing and autonomous decision-making (Schmidhuber, 2015; LeCun et al., 2015).

Research into modeling age-related changes in the brain using artificial neural networks and other computational approaches is still in relatively early stages. Although computational models have been used to model general brain processes, their application to age-related neural degeneration remains limited. However, it is a highly significant area of research for understanding the more intricate details of age-related decline of brain function particularly related to learning and memory consolidation. There remains a gap in research that is explicitly focused on modeling age-related changes in the brain, and it presents a critical opportunity for further research. In this paper, we present a computational model to mimic the learning and memory consolidation process of the brain. We model the learning process of young and old brains using a simple neural network with appropriate learning functions to simulate their behaviors. Through this model, we analyse the key differences as well as similarities between the function of young and old brains during learning.

## 2 Method

Our approach involves a simplified model of learning using an artificial neural network representing the neural connections (i.e., synapses) in the brain. The primary objective of our simulation is to model the number of learning iterations required by young and old brains to consolidate a memory. This section details the network structure and how we designed the learning task of the network.

### 2.1 Model structure

**Figure 1:**
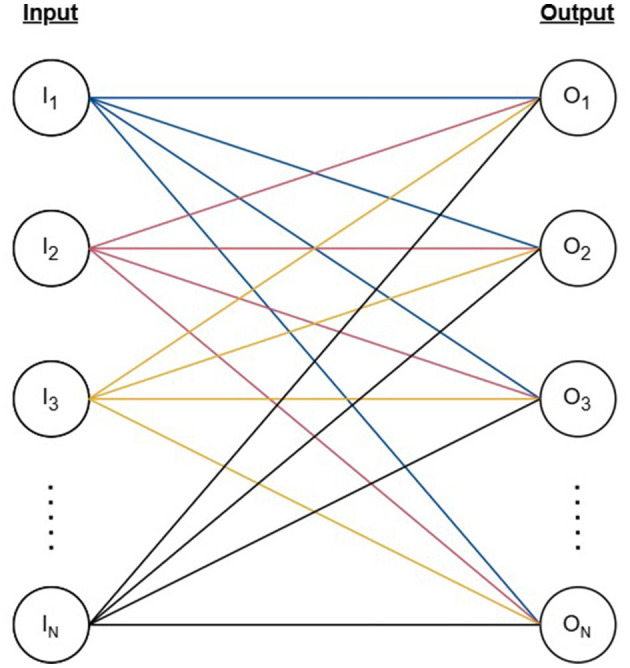
The structure of the artificial neural network represented by a bipartite graph with *N* vertices as input nodes, *N* vertices as output nodes, and *N*^2^ edges connecting each pair of input and output nodes.

Our model mimics the fundamental neural connectivity of the brain using an artificial neural network (ANN) designed to capture the biological learning mechanisms and memory consolidation techniques observed in neuroscientific studies. The model is structured as a fully connected ANN consisting of *N* binary input nodes (*I*) and *N* output nodes (*O*) connected by weighted edges (*E*) forming a bipartite graph as represented by Equation 1 and Figure 1. Similarly to conventional ANNs, the weights of our network activate and deactivate depending on the state of the inputs, and the signals reaching the output layer are adjusted accordingly. A firing threshold (*T*) selected based on the learning objective of the network determines the activation state of the output nodes.


(1) $$\begin{array}{l} G(V,E)\\ V=I\cup O\\ \;I\cap O=\emptyset \\ \;I=\{I_{i}:\;I_{i}\in \{0,1\}\}\\ O=\{O_{j}:\;O_{j}\in \mathbb{R}\}\\ E=\{(I_{i},\;O_{j},\;w_{ij}):\;I_{i}\in I,\;O_{j}\in O,\;w_{ij}\in \mathbb{R})\}\\ \;\forall i,j\in \{1,2,...,N\} \end{array}$$

The model we designed for our experiments consists of *N* = 30 input and output nodes. We initialized the weights of the model considering the baseline condition where all input nodes are active (*I*_*i*_ = 1 , ∀*i*∈{1, 2, ..., *N*}) resulting in a fully engaged network where signals flow through all edges. We initialized the weights for the edges by sampling from a normal distribution N(μ=1,σ=0.2) and rescaled the weights such that the sum of outgoing weights of each input node was equal to 1 corresponding to the activation status of the input (Equation 2).


(2) $$\begin{array}{l} w_{ij}\sim N(1,0.2)\\ w_{ij}\leftarrow \frac{w_{ij}}{\sum_{j=1}^{N}w_{ij}}\;,\;\forall i\in \{1,2,...,N\} \end{array}$$

Each output node accumulates the signals from all its incoming active edges as given by Equation 3, and under the initial model configuration where all input nodes are active, the weights of all incoming edges contribute to the output signal.


(3) $$\begin{array}{l} O_{j}=\overset{N}{\underset{i=1}{\sum }}I_{i}w_{ij}\;,\forall j\in \left\{1,2,...,N\right\} \end{array}$$

### 2.2 Input patterns and threshold generation

For our model, we considered input patterns where only six of the 30 input nodes are activated. This ratio was chosen as an appropriate one between activated and non-activated nodes based on the animal model in Tayler et al. (2013) where about 20% of the CA1 hippocampal neurons are involved in learning. Taking into account the possible combinations to activate six out of 30 nodes, there are 306 = 593,775 possible patterns that can be used for the experiments.

The activation of an input node indicates that all 30 outgoing edges of that specific node are activated and carry signal to the output nodes. To determine the overall signal reaching an output node, the weight of the six active incoming edges are summed for each node (Equation 3) and considering that the remaining 24 edges are from inactive input nodes, they are disregarded.

We determined the firing threshold (*T*) for the output signal in order to set up the model state before learning and reduce the total number of valid input patterns determined beforehand. We define the model state before the learning process as the input patterns where none of the output nodes are activated. That is, the signal reaching all output nodes are below the firing threshold. For our model, we determined *T* such that approximately only 25% of all input patterns are valid considering the model state before learning. Threshold values were hypothesized based on the range of maximum signal reaching an output node for the input patterns. The threshold found for the initial set of input patterns was 0.2405, which resulted in 137,017 valid input patterns, characterizing patterns before the learning process.^1^ Since valid input patterns are those with a maximum sum below the threshold, our model simulates a brain prior to a learning experience and will be further utilized to simulate different types of learning in young and old brains.

### 2.3 Learning process modeling

We modeled the learning process using two distinct learning rules for young and old brains which update the weights of edges corresponding to a chosen output node at each iteration or time step. We select output nodes to be updated using the roulette wheel selection to allow output nodes with higher signals more chance to activate. This procedure follows the fitness proportionate selection whereby a fitness is assigned to all output nodes based on its signal and the probability of each node *j* being selected is given by Equation 4. Therefore, the higher the signal of an output node, the higher its probability of undergoing the weight update.


(4) $$P(j)=\frac{O_{j}}{\sum_{k=1}^{N}O_{k}}\;,\;\forall j\in \{1,2,...,N\}$$

In each iteration we amend the sum of the signal reaching the selected output node based on the result of the weight updating rule, demonstrating the strengthening of the connections between input and output nodes. An output node is activated when the sum of its incoming activated edges surpasses the threshold *T* (*O*_*j*_≥*T*). We consider the learning process to be complete when six output nodes from each input pattern cross the threshold after undergoing the weight update process. The source code implementing the two learning approaches is publicly available on Github.^2^

In young learning, we update all incoming edges of the chosen output node in each iteration. We defined the weight updating rule for young learning as shown by Equation 5 where *w*_*ij*_ is the set of weights for all incoming active edges for the chosen output node *j*. Following the biological process of long-term potentiation (LTP), the function increases the strength of the weights at a constant learning rate α in each time step. In our experiments we used α = 1.5.


(5) $$\begin{array}{l} w_{ij}\leftarrow \alpha w_{ij}\;,\forall i\in \left\{1,2,...,N\right\} \end{array}$$

**Figure 2:**
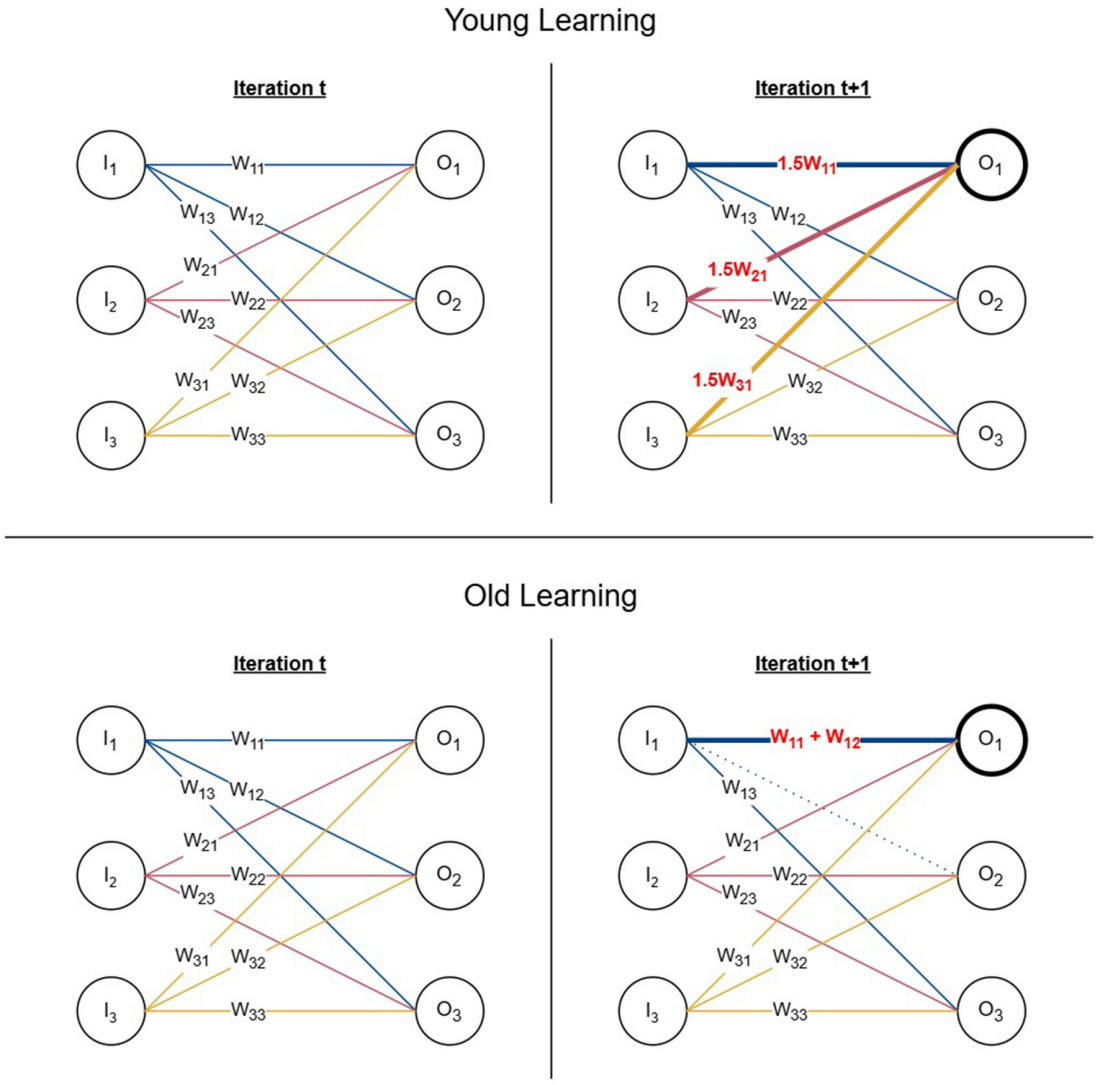
Young and old learning processes. **(Top)** One iteration of the young learning process represented using a 6-node network. The chosen output node is *O*_1_ and all its incoming edges are incremented by 0.5 of their initial weights resulting in the signal reaching the selected node being increased by 0.5 of its previous value. **(Bottom)** One iteration of the old learning process represented using a 6-node network. If the chosen output node is *O*_1_ and *w*_11_ is selected as the edge for the weight update, the second edge *w*_12_ is selected based on proximity from the same input node and its connection is re-wired from *O*_2_ to *O*_1_ by removing the edge and adding its weight to *w*_11_.

We repeat the weight updating process by selecting output nodes using the roulette wheel selection until the signal of six output nodes surpass the threshold. Figure 2 (top) demonstrates one iteration of the young learning process using a simplified neural network with six nodes. If node with signal *O*_1_ is chosen by the roulette wheel selection, all its incoming edges are updated by $\frac{1}{2}$ of their previous weights which also increments the signal *O*_1_ by $\frac{1}{2}$ its previous value.

In old learning, we update only one edge in a single iteration. We defined the weight update function for old learning as shown by Equation 6 where *w*_*ij*_ is the weight of the edge to be updated and *w*_*ik*_ being an active outgoing edge of the same input node *i*.


(6) $$\begin{array}{l} w_{ij}\leftarrow w_{ij}+w_{ik}\\ w_{ik}\leftarrow 0 \end{array}$$

Old learning is modeled based on the concept of multi-innervated dendritic spines (MIS) where synaptic connections are strengthened by attracting additional synapses. Once an output node is chosen to be updated using roulette wheel selection, two edges are selected to perform MIS. The first edge (*w*_*ij*_) is chosen using roulette wheel selection similarly to Equation 4, however, using the magnitude of the weights rather than output signals. It is reinforced with the weight of the second edge (*w*_*ik*_), selected using anatomical proximity with coin tossing simulation to determine the direction in which it would be chosen relative to the input node. The second edge (*w*_*ik*_) is chosen from the same input node *i* where the first edge (*w*_*ij*_) originates. This is to represent the MIS process, which is formed of two presynaptic neurons and one postsynaptic neuron. We remove the existing connection of the second edge (*w*_*ik*_) and add its weight to the first edge (*w*_*ij*_) simulating the behavior of re-wiring connections. If the weight updating procedure does not push the output signal over the threshold in the current iteration, we select the next edge based on proximity as *w*_*ik*_ and add its weight to the updated first edge *w*_*ij*_ in the next iteration. We repeat the process until the signal crosses the threshold or all possible edges are added to the first edge. In the latter scenario, we select the next output node according to the roulette wheel selection to continue the learning process from the next iteration. Figure 2 (bottom) represents one iteration of the old learning process using a simplified network of six nodes. When *O*_1_ and *w*_11_ are chosen using the roulette wheel selection as the output node and its edge to be updated, a second edge is selected from the remaining outgoing edges of the same input node with signal *I*_1_ based on proximity. Assuming that the selected second edge is *w*_12_, it is re-wired to *O*_1_ by removing its original connection from *O*_2_ and adding its weight to *O*_1_.

## 3 Results

This section presents an analysis of the learning processes and output patterns generated by our model under young and old learning conditions, highlighting differences in learning speed, output similarity and memory specificity.

**Figure 3:**
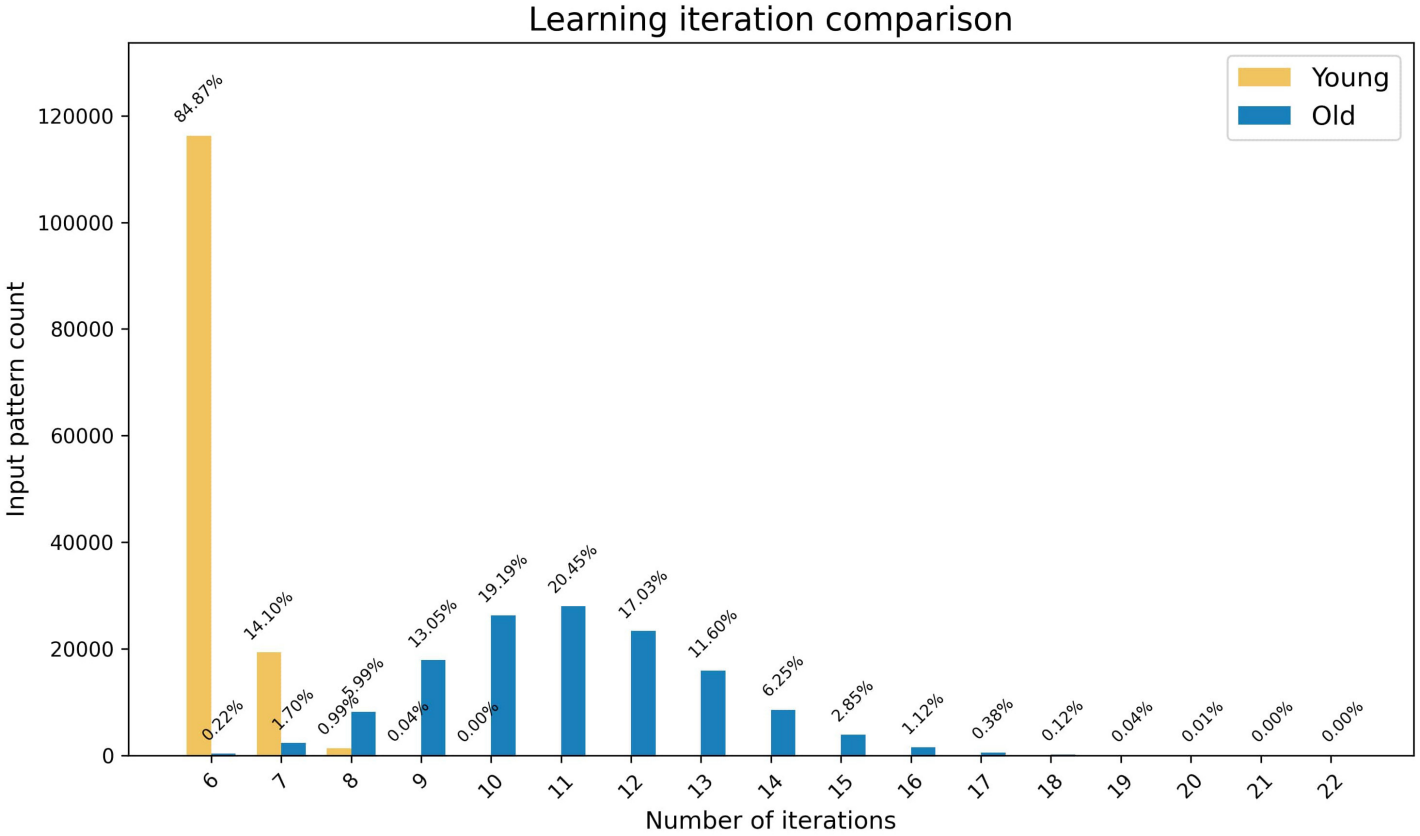
The graph represents the comparison of speed of the learning processes in young and old brains. The x-axis shows the number of iterations and the y-axis shows the number of patterns that completed learning at each iteration, with percentages highlighted as data tips.

Considering that both young and old learning update the weights corresponding to one output node at any given time step, a minimum of six iterations are required for any input pattern to complete learning for both methods. As shown in Figure 3, the young learning process completes in the minimum number of required iterations for ~85% of input patterns. This indicates that for these patterns each selected output node underwent the weight update process only once for its signal to surpass the firing threshold. The remaining patterns completed learning in under 10 iterations. In contrast, only 0.2% of the patterns complete the old learning process in six iterations, with the remaining patterns taking up to 22 iterations to complete. These results indicate that the model along with the biologically inspired weight update rules effectively simulates the learning speed of young and older brains with young learning being considerably faster.

In addition to the comparison of learning speeds, we analyzed the similarity of output patterns obtained via young and old learning for the same inputs. We define the similarity between two patterns *P*_*a*_ and *P*_*b*_ as the number of common active nodes.

We observed that there are no patterns that result in identical output patterns (i.e., all six active nodes are the same) for young and old learning. Meanwhile, 36 patterns yield outputs with five common active nodes which accounts for less than 0.1% of all patterns. More than 80% of the output patterns have two or fewer active nodes in common. This outcome indicates the tendency of young and old learning mechanisms to activate different neuron patterns when learning the same concepts.

Additionally, we investigated the specificity of the output patterns generated by young and old learning. We observed that ~79% of the output patterns are unique in both categories. Among the remaining patterns, 9–10% of have two copies and ~0.75% have three copies. Furthermore, there are 58 and 54 patterns with four copies for young and old learning respectively, while one and two patterns have five copies which accounts for ~0.04% of all the output patterns. This high specificity indicates that despite some replication, the output patterns generated by both young and old learning remain largely distinct. Moreover, the activation probability of output nodes follows a near-uniform distribution, with all nodes having a probability of activation within the range of 17%–21%, and a maximum difference of 1% between young and old learning.

Although both young and old learning exhibit the same trend in output pattern specificity, we observed that the two groups of output patterns are largely disjoint. Only 25,527 output patterns are common between young and old learning, accounting for ~21% of distinct patterns. This indicates that although the specificity of memory can be preserved in older brains, the formation of memory varies due to the differences in learning mechanisms.

**Figure 4:**
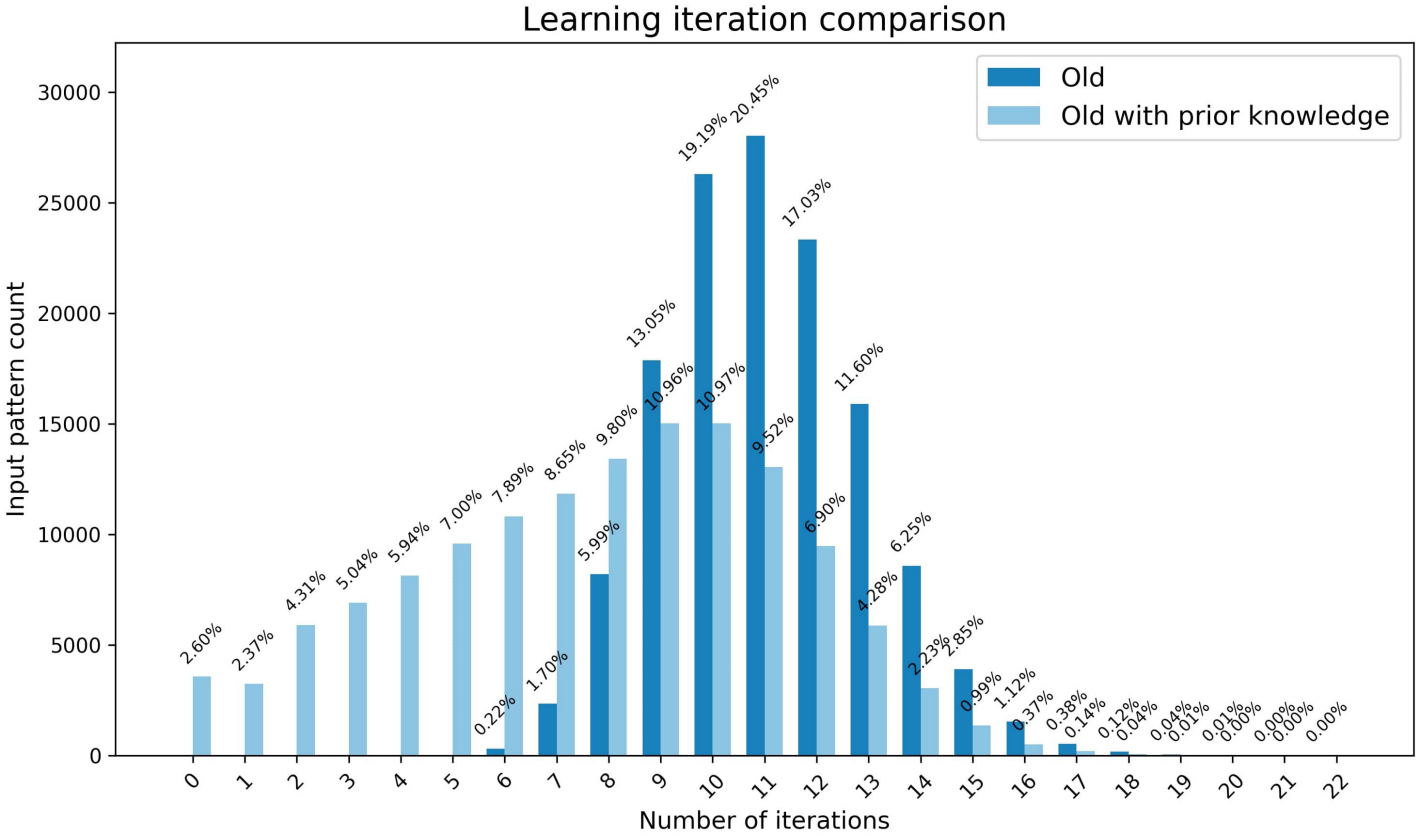
The graph represents the comparison of speed of the learning in old brains with and without prior learning. The x-axis shows the number of iterations and the y-axis shows the number of patterns that completed learning at each iteration, with percentages highlighted as data tips.

In our preliminary experiment, we used the clean-slate assumption for both young and old learning where both started with a state where no output nodes were activated for each input pattern assuming that no prior learning has taken place. However, older brains often benefit from previously accumulated knowledge for learning new concepts. Therefore, we compared the speed of old learning with and without prior knowledge using weights preloaded by young learning for one input pattern and the initial weights respectively. As demonstrated by Figure 4, there is significant speed up in learning with prior knowledge. More than 2% of the output patterns are at a learned state from the start which results in not requiring any iterations to complete learning. Approximately 27% of the patterns require less than six iterations indicating that they had at least one active output node prior to learning.

## 4 Discussion

In this study our aim was to develop a computational model that captures the biological mechanisms underlying the learning processes in young and ageing brains. To achieve this, we constructed a simple artificial neural network using a bipartite graph, with nodes and edges representing the neurons and their synapses, respectively. We generated input patterns where six out of 30 nodes are activated and established a threshold to use 25% of patterns as our “before learning” phase. We simulated the learning process by updating edge weights until six output nodes received a signal greater than the threshold, which characterized the completion of learning. We used a learning rule inspired by long-term potentiation for young learning and a rule based on multi-innervated synapses for old learning.

Our results demonstrate that the learning mechanism of younger brains take less time for learning compared to older brains when starting with the same initial conditions, which coincides with the natural biological observations of the human brain function and behavior. This suggests that our model effectively mirrors the fundamental aspects of learning speed and memory consolidation observed in the brain. Additionally, our findings indicate that young and old learning processes tend to utilise different spatial locations for memory storage, while maintaining a high degree of memory specificity that follows similar trends across age groups. Furthermore, our results demonstrate that old learning with prior knowledge is considerably quicker compared to learning with a clean slate which gives insights to the efficiency of building on previously stored knowledge compared to acquiring completely new information.

Various biologically inspired models of learning can be found in previous studies. Particularly, restricted Boltzmann machines (RBMs) (Hinton and Salakhutdinov, 2006) share architectural similarities with our bipartite graph-based model, as both use two-layer structures with weighted connections. However, our approach differs by incorporating biologically motivated learning rules inspired by long-term potentiation (LTP) and multi-innervated synapses (MIS), focusing on synaptic modifications in young and ageing brains rather than probabilistic inference or energy minimisation.

A crucial future direction in our research involves the analysis of memory capacity and the spatial locations of memory consolidation in young and ageing brains. The hippocampus and prefrontal cortex are known to play vital roles in memory formation and retrieval, but these regions undergo significant changes with age which leads to decline in memory capacity. This analysis is important because memory is not stored uniformly across the brain and different types of memory may be affected to varying degrees by ageing. In neural networks, memory capacity is bound by the plasticity-stability trade-off where the increase of the ability to learn new memories leads to faster forgetting of old ones. By integrating findings on synaptic complexity and memory representation within neural network models, future studies could provide deeper insights into how memory capacity diminishes with age and how this process might be mitigated.

## Data Availability

The datasets presented in this study can be found in online repositories. The names of the repository/repositories and accession number(s) can be found in the article/supplementary material.

## Footnotes

^1^The datasets generated for this study can be found in the King's College London research data repository, KORDS at https://doi.org/10.18742/28143317.

^2^ https://github.com/JayaniH/ageing-brains-learning-models/tree/main
